# Comparison of pancreatic lipase inhibitory isoflavonoids from unripe and ripe fruits of *Cudrania tricuspidata*

**DOI:** 10.1371/journal.pone.0172069

**Published:** 2017-03-02

**Authors:** Yang Hee Jo, Seon Beom Kim, Qing Liu, Seon-Gil Do, Bang Yeon Hwang, Mi Kyeong Lee

**Affiliations:** 1 College of Pharmacy, Chungbuk National University, Cheongju, Chungbuk, Republic of Korea; 2 Wellness R&D Center, Univera, Inc., Seoul, Republic of Korea; The University of Tokyo, JAPAN

## Abstract

The composition and content of the active constituents and their biological activity vary according to diverse factors including their maturation stages. A previous study showed that the fruits of *Cudrania tricuspidata* inhibited pancreatic lipase activity, a key enzyme in fat absorption. In this study, we investigated the chemical composition and pancreatic lipase inhibitory activity of unripe and ripe fruits of *C*. *tricuspidata*. Unripe fruits of *C*. *tricuspidata* have a higher content of total phenolic and flavonoids and exhibited stronger pancreatic lipase inhibition compared to ripe fruits. HPLC analysis revealed the different chemical compositions of the unripe and ripe fruits. Further fractionation resulted in the isolation of 30 compounds including two new isoflavonoids. Analysis of the chemical constituents of the unripe and ripe fruits revealed that a 2,2-dimethylpyran ring, a cyclized prenyl, was the predominant side chain in the unripe fruits, whereas it was a linear prenyl group in the ripe fruits. In addition, a new isoflavonoid (**19**) from the unripe fruits showed the most potent inhibition on pancreatic lipase. Taken together, the maturation stage is an important factor for maximum efficacy and that unripe fruits of *C*. *tricuspidata* are a good source of new bioactive constituents for the regulation of obesity

## Introduction

A global rise in obesity has become a widespread issue due to its association with diverse pathological disorders, including atherosclerosis, diabetes, hypertension, and cancer [1,2]. High consumption of saturated fats in Western diets is suggested as one of the main contributors to obesity, as demonstrated by the correlation between the amount of dietary fat and obesity in epidemiological study. Fat is an ester of three fatty acid and glycerol and absorbed after digestion into monoglyceride and fatty acids by lipase. Lipase is a key enzyme in lipid absorption and pancreatic lipase, a main lipase of the human, is responsible for the hydrolysis of 50–70% of total dietary fats. Therefore, a reduction in fat absorption by the inhibition of pancreatic lipase is suggested to be beneficial for the regulation of obesity [3–4]. A specific pancreatic lipase inhibitor, orlistat, has been clinically used for the prevention of obesity [5–6] and many fields of research have focused on developing anti-obesity therapeutics with more efficiency and less side effect. In particular, food and food ingredients are considered good targets for anti-obesity agents to prevent obesity and obesity-associated disorders [7–9].

*Cudrania tricuspidata* (Moraceae family), is a thorny tree cultivated in East Asia including Korea. The fruits of *C*. *tricuspidata*, a kind of berry, are consumed as fresh fruits, juices and jams. The fruits of *C*. *tricuspidata* are also used in processed products such as wine and vinegar are also available. The fruits of *C*. *tricuspidata* are rich in diverse active constituents. Polyphenols, such as flavonoids, possess diverse biological activities (e.g., antioxidant, estrogenic, anti-inflammatory and anti-cancer activity) and are considered major functional components [10–13]. As a result, the utility of this fruit as an ingredient in dietary supplements and functional foods ingredients is being actively investigated in many fields.

The composition and content of flavonoids vary, depending on environmental conditions during cultivation [14]. Temperature, humidity, insects and agricultural chemicals affect the content of phytochemicals. Maturation is one of the main factors that influence the composition and content of active constituents [15–17]. Therefore, focusing on different maturation stages of fruits may help uncover new bioactive constituents.

We recently reported the pancreatic lipase inhibitory activity of ethanol extract of *C*. *tricuspidata* fruits and suggested that 6,8-diprenylgenistein, a major isoflavonoid, was an active constituent [18]. Anti-obesity effect of 6,8-diprenylgenistein was also demonstrated in high-fat diet-induced obese mice [19]. In a continuation of our investigation about the anti-obesity effect of *C*. *tricuspidata* fruit, we conducted further studies to elucidate active constituents of unripe and ripe fruits. In the present study, we describe the structure of new compounds and the pancreatic lipase activity of constituents of unripe and ripe fruits. The effect of maturity on the chemical constituents and biological activity is also elucidated.

## Materials and methods

### General experimental

A JASCO DIP-1000 polarimeter was used for the measurement of optical rotations. A JASCO UV-550 and Perkin-Elmer model LE599 spectrometer were used respectively, for the measurement of UV and IR spectra. NMR spectra were recorded on a Bruker DRX 400, 500 or 700 MHz spectrometer using methanol-*d*_4_ or acetone-*d*_*6*_ as solvents. ESIMS data was obtained on VG Autospec Ultima mass spectrometers.

### Plant materials

**Fig 1:**
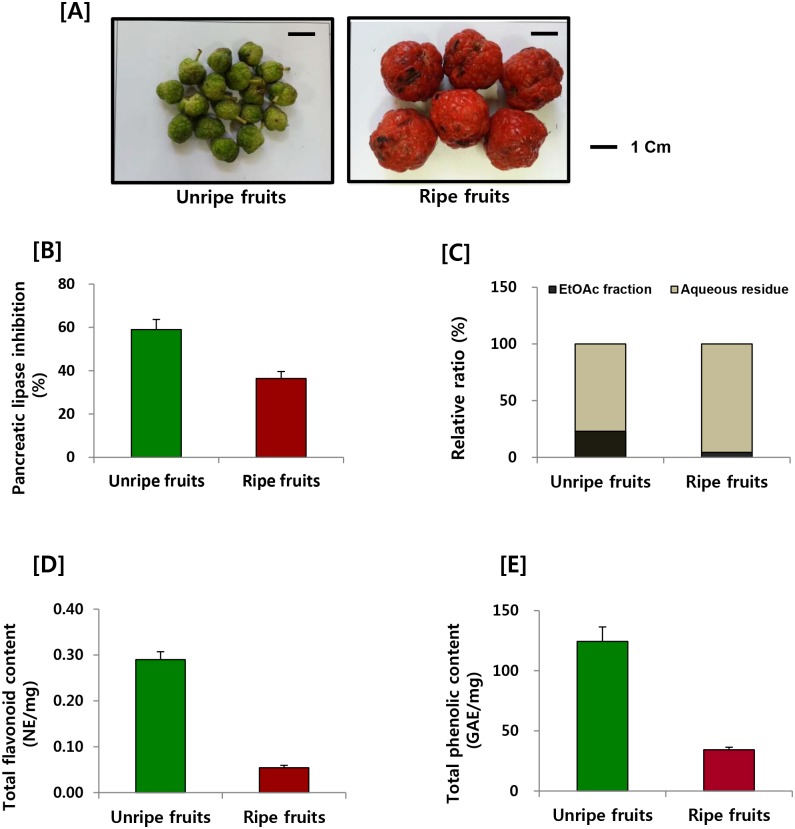
(A) Photographs of unripe and ripe fruits of *C*. *tricuspidata*, (B) effect on pancreatic lipase activity, (C) relative ratio of EtOAc and aqueous fraction, (D) effect on total flavonoid contents, and (E) effect on total phenolic content.

The fruits of *C*. *tricuspidata* were collected from the herb garden at Chungbuk National University from September to October 2013. After identification of the fruits by the herbarium of the College of Pharmacy, Chungbuk National University, the fruits were divided into unripe and ripe depending on color (Fig 1A). Voucher specimens for the unripe fruits (CBNU2013-CTUF) and ripe fruits (CBNU2013-CTRF) were deposited in a specimen room of the herbarium.

### Measurement of fruit color

The colors of *C*. *tricuspidata* fruits were measured using a CR-400 colorimeter. Values of L*, a* and b* used to define the colors. L* indicates lightness (0 for opaque or black and 100 for transparent or white), a* indicates redness (+ a* for redness and—a* for greenness), and b* indicates yellowness (+ b* for yellowness and—b* for blueness) [20].

### Preparation of unripe fruits and ripe fruits extract

For the preparation of extract, 1 g of powdered unripe and ripe fruit of *C*. *tricuspidata* was extracted with 10 ml of methanol, respectively. The filtrate was evaporated *in vacuo*, yielding unripe and ripe fruit extract for further investigation. For the preparation of EtOAc soluble fraction, 10 mg of unripe fruits and ripe fruits extract was suspended, respectively, in 1 ml of water. Then, 1 ml of EtOAc was added to each mixture and mixed vigorously. After centrifugation of mixture for 30 s, EtOAc fraction and water fraction were collected, respectively.

### Measurement of total flavonoid content

The total flavonoid content was measured with an aluminum chloride colorimetric assay. Samples were added to a 96-well plate, followed by the addition of 5% NaNO_3_. After 5 min incubation, 10% AlCl_3_ was added to the reaction mixture. After incubation with gentle shaking, 1 N NaOH and H_2_O were added to the reaction mixture. The absorbance was measured at 510 nm with a microplate reader. The total flavonoid content was expressed as catechin equivalent (NE) using catechin as a standard.

### Measurement of total phenolic content

The total phenolic content was measured with a Folin-Ciocalteau assay. Folin-Ciocalteau’s phenol reagent was added to the 96-well plate containing the test samples. After 5 min of incubation with gentle shaking, 7% Na_2_CO_3_ was added to the reaction mixture. The reaction mixture was left in the dark for 90 min at room temperature. The absorbance was measured at 630 nm with a microplate reader. The total phenolic content was expressed as gallic acid equivalent (GAE) using gallic acid as a standard.

### HPLC analysis

Unripe and ripe fruit samples were prepared in methanol at a concentration of 10.0 mg/ml. Each sample solution was filtered through a 0.45 μm membrane filter before HPLC analysis. HPLC analysis was performed using a Waters HPLC system equipped with Waters 600 Q-pumps, a 996 photodiode array detector, and Waters Empower software using Phenomenex Gemini-NX 3μ C18 110A (150 x 4.60 mm) for quantitation. Chromatographic separation was accomplished using acetonitrile-water (60:40) at a flow rate of 1.0 mL/min. The wavelengths for the detection and retention time were set at 254 nm and 16 min, respectively.

### Pancreatic lipase activity

Pancreatic lipase activity was determined by measuring the hydrolysis of *p*-nitrophenyl butyrate (*p*-NPB) to *p*-nitrophenol using a method reported previously [18]. The 0.1 mg/ml of enzyme solution was prepared by reconstituting porcine pancreatic lipase using 0.1 M Tris-HCl buffer (pH 8). Then, 5 μl of test sample was mixed with 90 μl of enzyme buffer, and incubated for 15 min at 37°C. After incubation, 5 μl of 10 mM *p*-nitrophenylbutyrate (*p*-NPB) was added to enzyme mixture and the reaction was allowed to proceed for further 15 min at 37°C. After incubation, the absorbance was measured at 405 nm using a microplate reader. Relative pancreatic lipase activity (%) was calculated as [(the activity of the compound with the substrate—the activity of the compound without the substrate)/(activity without the compound and with the substrate—negative control without the compound and substrate)] x 100. Orlistat was used as a positive control.

Pancreatic lipase activity against triglyceride was also determined using Lipase Activity Assay Kit according to manufacturer’s protocol (Sigma-Aldrich Co.). Relative pancreatic lipase activity (%) was calculated using glycerol as a standard.

### Statistical analysis

The evaluation of statistical significance was determined by a one-way ANOVA test, with a value of *p*<0.05 or less considered to be statistically significant.

## Results and discussion

### Effects of maturity

#### Division into unripe and ripe groups

The fruits of *C*. *tricuspidata* were collected at different maturation stages and divided into two groups, unripe (green) and ripe (red) fruits, by their color appearance and color-difference meter (Table 1). The sizes of ripe fruits were much bigger than unripe ones, as determined by weight and diameter. The a* value which determines red (+) or green (-) were significantly different between two groups. The b* value which determines yellow (+) or blue (-) were also different between two groups, whereas little differences of the L* values, which indicated the lightness.

**Table 1:** Weight, size and values of L*, a*, b* of ripe and unripe fruits of *C*. *tricuspidata*.

	Weight	diameter	L*	a*	b*
Unripe fruits	0.43 ± 0.07	1.01 ± 0.08	34.51 ± 0.25	-4.28 ± 0.53	8.62 ± 0.58
Ripe fruits	13.3± 2.88	3.05 ± 0.37	33.41 ± 0.98	26.80 ± 0.09	16.57 ± 0.71

#### Effect on chemical composition and pancreatic lipase inhibition

**Fig 2:**
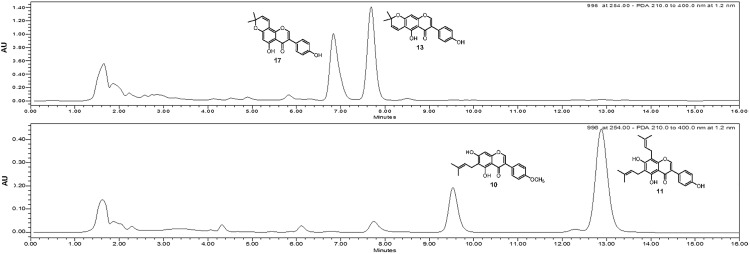
HPLC chromatogram of extract from (A) unripe and (B) ripe fruits. Chromatographic separation was accomplished by Gemini-NX 3μ C18 110A (150 x 4.60 mm) using acetonitrile-water (60:40) at a flow rate of 1.0 mL/min. The wavelengths for the detection was set at 254 nm.

To investigate the effect of the maturity of *C*. *tricuspidata* fruits on their chemical composition and biological activity, they were divided into unripe and ripe two groups”`(Fig 1A). First, we determined the pancreatic lipase inhibitory activity of unripe and ripe fruits. The inhibitory activity of the total extract of the unripe fruits was stronger than that of the ripe fruits at 100 μg/ml (Fig 1B). Next, we compared the chemical composition of the unripe and ripe fruits. The water-soluble fraction of the ripe fruit extract was relatively higher than that of the unripe fruits extract (Fig 1C). The flavonoid and phenolic content was much higher in the unripe fruit extract (Fig 1D and 1E). Further HPLC analysis confirmed the difference in the major constituents of the unripe and ripe extract (Fig 2).

Taken together, there was a substantial difference in the chemical composition of the unripe and ripe fruits. The phenolic and flavonoid content was higher in the unripe fruit and lower in the ripe fruit (Fig 1D and 1E). The increase in the dry weight due of the accumulation of sugars and anthocyanins might explain this finding. The red color and the increase of water-soluble fraction in the ripe fruit extracts provide further support for this idea (Fig 1A and 1C). The differences in the chemical composition affected the pancreatic lipase activity, with the unripe fruit extract showing stronger pancreatic lipase inhibition compared to that the ripe fruit extract (Fig 1C). Therefore, the maturation stages of *C*. *tricuspidata* fruits were suggested to be an important factor for active resources.

### Identification of compounds

#### Isolation of compounds from unripe and ripe fruits

As the HPLC patterns of the unripe and ripe fruits extract of *C*. *tricuspidata* were very different, further separation of each unripe and ripe fruit extract was carried out to elucidate the difference in the constituents, depending on maturity.

The unripe fruits of *C*. *tricuspidata* (556.0 g) were extracted twice with 100% MeOH, which yielded the methanol extract (20.4 g). The methanol extract was suspended in H_2_O and partitioned successively with *n*-hexane (0.8 L), CH_2_Cl_2_ (0.7 L), EtOAc (0.8 L), and *n*-BuOH (0.8 L). The CH_2_Cl_2_ fraction of the unripe fruits (CTUM, 3.3 g) was subjected to Sephadex LH-20 and eluted with MeOH to give four subfractions (CTUM1-CTUM4). The CTUM2 was subjected to medium-pressure liquid chromatography (MPLC) over silica gel and eluted with hexane-EtOAc (20:1) to give six subfractions (CTUM2A-CTUM2F). Compounds **14** (17.3 mg), **18** (6.6 mg), **21** (3.2 mg), and **22** (2.8 mg) were obtained from CTUM2B by semi-preparative HPLC eluting with MeCN-water (80:20). The CTUM3 was purified by semi-preparative HPLC with MeCN-water (57:43) to give compounds **13** (278.2 mg), **15** (2.6 mg), **17** (111.1 mg), and **19** (2.2 mg). The EtOAc fraction of unripe fruits (CTUE, 0.4 g) was subjected to silica gel MPLC and eluted with CH_2_Cl_2_-MeOH (50:1) to yield five subfractions (CTUE1-CTUE5). Compounds **6** (0.6 mg) and **7** (0.9 mg) were obtained from CTUE2 by semi-preparative HPLC eluting with MeCN-water (20:80).

The ripe fruits of *C*. *tricuspidata* (600 g) were extracted twice with 100% MeOH, which yielded methanol extract (247.0 g). The methanol extract was suspended in H_2_O and partitioned successively with *n*-hexane (3.7 L), CH_2_Cl_2_ (3.0 L), EtOAc (3.7 L), and *n*-BuOH (3.7 L). The CH_2_Cl_2_ fraction of red fruits (CTRM, 7.2 g) was subjected to MPLC over silica gel and eluted with *n*-hexane-EtOAc (20:1) to give 12 subfractions (CTRM1-CTRM12). The CTRM6 fraction was subjected to Sephadex LH-20 and eluted with CH_2_Cl_2_-MeOH (1:1) to afford two subfractions (CTRM6A-CTRM6B). Compounds **3** (0.9 mg), **16** (1.6 mg), and **20** (0.8 mg) were obtained from CTRM6B by semi-preparative HPLC eluting with MeCN-water (80:20). Compound **11** (926.5 mg) was purified from CTRM7 by recrystallization from *n*-hexane-CH_2_Cl_2_ (1:1). Compound **10** (525.7 mg) was obtained by recrystallization of CTRM8 with *n*-hexane-CH_2_Cl_2_ (1:1). CTRM10 was subjected to Sephadex LH-20 and eluted with CH_2_Cl_2_-MeOH (1:1) to yield five subfractions (CTRM10A-CTRM10E). Compounds **9** (1.8 mg) and **12** (2.9 mg) were obtained from CTRM10D by semi-preparative HPLC eluting with MeCN-water (80:20). CTRM11 was chromatographed over Sephadex LH-20 with CH_2_Cl_2_-MeOH (1:1) to give Compound **1** (3.1 mg). The EtOAc fraction of the ripe fruits (CTRE, 2.2 g) was subjected to RP-MPLC and eluted with MeOH-H_2_O (1:5) to yield five subfractions (CTRE1-CTRE5). The CTRE1 was rechromatographed on Sephadex LH-20 using MeOH to yield five subfractions (CTRE1A-CTRE1E). The CTFE1B was further separated by RP-MPLC with MeOH-H_2_O (1:5) to give three subfractions (CTRE1B1-CTRE1B3). Compound **25** (31.2 mg) was obtained from CTRE1B1 by semi-preparative HPLC eluting with MeCN-water (15:85). Compounds **4** (2.1 mg), **8** (4.8 mg) and **30** (1.7 mg) were obtained from CTRE1B2 by semi-preparative HPLC eluting with MeCN-water (20:80). Compounds **26** (6.9 mg) and **29** (1.9 mg) were obtained from CTRE1B3 by semi-preparative HPLC eluting with MeCN-water (20:80). Compounds **5** (0.8 mg), **23** (10.8 mg), **24** (7.1 mg), **27** (5.1 mg), and **28** (9.3 mg) were obtained from CTRE1C by semi-preparative HPLC eluting with MeCN-water (20:80). Compound **2** (3.3 mg) was purified from CTRE1E by recrystallization using MeOH.

**Cudracusisoflavone A (7).** Brown amorphous gum; UV (MeOH) λ_max_ 261 nm; IR_max_ 3298, 1415 cm^-1^; ^1^H NMR (CD_3_OD, 400 MHz) δ 8.17 (1H, s, H-2), 7.27 (1H, d, *J* = 2.0 Hz, H-2'), 7.25 (1H, d, *J* = 8.4 Hz, H-5'), 7.10 (1H, dd, *J* = 8.4, 2.0 Hz, H-6'), 6.38 (1H, d, *J* = 2.0 Hz, H-8), 6.25 (1H, d, *J* = 2.0 Hz, H-6), 4.97 (1H, d, *J* = 6.8 Hz, H-1''), 3.93 (3H, s, OCH_3_), 3.75~3.44 (5H, m, H-2'', 3'', 4'', 5'', 6'') ppm; ^13^C NMR (CD_3_OD, 125 MHz) δ 180.6 (C-4), 164.8 (C-5), 162.5 (C-7), 158.3 (C-8a), 154.0 (C-2), 149.2 (C-3'), 146.7 (C-4'), 125.8 (C-1'), 122.9 (C-3), 121.4 (C-6'), 116.5 (C-5'), 113.5 (C-2'), (C-4a), 101.3 (C-1''), 98.9 (C-6), 93.5 (C-8), 76.9 (C-5''), 76.5 (C-3''), 73.5 (C-2''), 70.0 (C-4''), 61.1 (C-6''), 55.4 (OCH_3_) ppm; ESIMS (negative mode) *m/z*: 461 [M-H]^-^; HRESI-MS (positive mode) *m/z*: 485.1067 (calcd for C_22_H_22_NaO_11_ 485.1060).

**Cudracusisoflavone B (19).** Brown amorphous gum; UV (MeOH) λ_max_ 269 nm; IR_max_ 3260, 1647 cm^-1^; ^1^H NMR (acetone-*d*_*6*_, 400 MHz) δ 13.17 (1H, s, 5-OH), 8.25 (1H, s, H-2), 7.17 (1H, d, *J* = 2.0 Hz, H-2'), 6.97 (1H, dd, *J* = 8.4, 2.0 Hz, H-6'), 6.90 (1H, d, *J* = 8.0 Hz, H-5'), 6.74 (1H, d, *J* = 10.0 Hz, H-1''), 6.21 (1H, s, H-6), 5.77 (1H, d, *J* = 10.0 Hz, H-2''), 1.49 (6H, s, CH_3_-4'', 5'') ppm; ^13^C NMR (acetone-*d*_*6*_, 100 MHz) δ 181.0 (C-4), 162.4 (C-5), 159.4 (C-7), 153.5 (C-2), 152.1 (C-8a), 145.4 (C-4'), 144.7 (C-3'), 127.8 (C-2''), 123.4 (C-3), 122.6 (C-1'), 120.7 (C-6'), 116.4 (C-2'), 115.1 (C-5''), 114.2 (C-1''), 105.8 (C-4a), 101.0 (C-8), 99.5 (C-6), 78.1 (C-3''), 27.4 (C-4'', 5'') ppm; ESIMS (positive mode) *m/z*: 353 [M+H]^+^; HRESIMS (positive mode) *m/z*: 375.0850 (calcd for C_20_H_16_NaO_6_ 375.0845).

#### Structural determination of the new compounds

**Fig 3:**
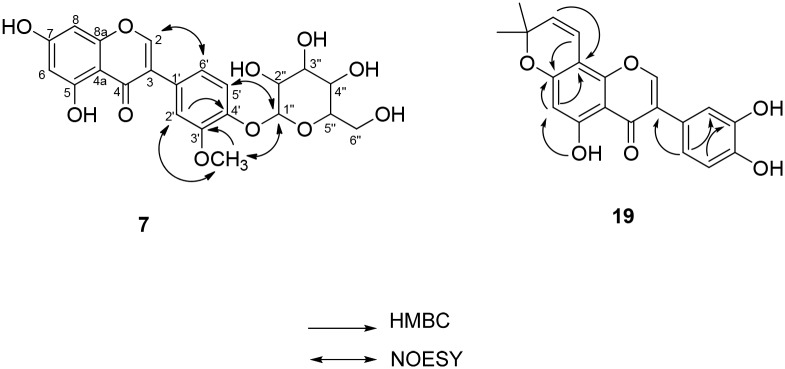
Key HMBC correlation of compounds 7 and 19 from unripe fruits of *C*. *tricuspidata*.

Compound **7** was obtained as brown amorphous gum and gave a pseudomolecular ion [M+Na]^+^ by HREIMS at 485.1067 (calcd 485.1060), consistent with a molecular formula of C_22_H_22_O_11_. Compound **7** was supposed to be an isoflavonoid from the signal at 8.17 (1H, s, H-2) in the ^1^H NMR spectrum. The ^1^H NMR spectrum also revealed the signals of a 1,3,4-trisubstituted aromatic ring at [δ_H_ 7.10 (1H, dd, *J* = 8.4, 2.0 Hz, H-6'), 7.25 (1H, d, *J* = 8.4 Hz, H-5') and 7.27 (1H, d, *J* = 2.0 Hz, H-2')] and 1,3,4,5-tetrasubstituted aromatic ring at [δ_H_ 6.25 (1H, d, *J* = 2.0 Hz, H-6) and 6.38 (1H, d, *J* = 2.0 Hz, H-8)]. The ^1^H and ^13^C NMR spectrum also indicated the presence of a glucose moiety from the signals at [δ_H_ 4.97 (1H, d, *J* = 6.8 Hz, H-1''); δ_C_ 101.3 (C-1''), 73.5 (C-2''), 76.5 (C-3''), 70.0 (C-4''), 76.9 (C-5'') and 61.1 (C-6'')]. The presence of a methoxyl group was deduced from the signals at δ_H_ 3.93 (3H, s) connected to δ_C_ 55.4 in the HSQC spectrum. Based on the aforementioned finding, compound **7** seems to be an isoflavonoid glycoside with a methoxyl group. The position of the methoxyl group was deduced to be C-3' from the HMBC correlation between δ_H_ 3.93 (OCH_3_) and δ_c_ 149.2 (C-3') (Fig 3). The position of the glucose moiety was assigned to C-4' from the NOESY correlation between δ_H_ 4.97 (H-1'') and δ_H_ 7.25 (H-5'), δ_H_ 3.93 (3'-OCH_3_). Therefore, compound **7** was determined as shown in Fig 3 and named cudracusisoflavone A.

Compound **19** was obtained as brown amorphous gum and gave a pseudomolecular ion [M+Na]^+^ by HREIMS at 375.0850 (calcd 375.0845), consistent with a molecular formula of C_20_H_16_O_6_. Similar to compound **7**, the characteristic signal for isoflavonoid at 8.25 (1H, s, H-2) was observed in the ^1^H NMR spectrum. The presence of a 2,2-dimethylpyran ring was deduced from the signals at [δ_H_ 6.74 (1H, d, *J* = 10.0 Hz, H-1''), 5.77 (1H, d, *J* = 10.0 Hz, H-2'') and 1.49 (6H, s, H-4''.5''); δ_C_ 114.2 (C-1''), 127.8 (C-2''), 78.1 (C-3''), and 27.4 (C-4'',5'')] in the ^1^H and ^13^C NMR spectrum, respectively, which is confirmed by HMBC correlations. The presence of an additional 1,3,4-trisubstituted aromatic ring was suggested from the signals at [δ_H_ 7.17 (1H, d, *J* = 2.0 Hz, H-2'), 6.90 (1H, d, *J* = 8.0 Hz, H-5') and 6.97 (1H, dd, *J* = 8.4, 2.0 Hz, H-6')] in the ^1^H NMR spectrum. The HMBC correlation between δ_H_ 6.97 (H-6') and δ_C_ 123.4 (C-3), 116.4 (C-2'), and between δ_H_ 6.90 (H-5') and δ_C_ 144.7 (C-3') confirmed the position of hydroxyl groups as C-3' and C-4' of B ring. The position of a 2,2-dimethylpyran ring was also determined from the HMBC correlation between H-1'' (δ_H_ 6.74) and C-7 (δ_C_ 159.4) and between H-2'' (δ_H_ 5.77) and C-8 (δ_C_ 101.0) to C-7 and C-8 (Fig 3). Based on these data, compound **19** was determined as shown in Fig 3. Wei et al [21] reported the same structure as 5,3',4'-trihydroxy-2", 2"-dimethylpyrano (5",6":7,8) isoflavone, however, the NMR data were quite different from those of compound **19** but similar to those of parvisoflavones A [21]. Especially, the ^1^H NMR data of B ring of 5,3',4'-trihydroxy-2",2"-dimethylpyrano (5",6":7,8) isoflavone [δ_H_ 6.39 (H-2'), 7.03 (H-5') and 6.37 (H-6')] of are different from those of compound **19** [δ_H_ 7.17 (H-2'), 6.90 (H-5') and 6.97 (H-6')] but almost identical to those of parvisoflavones A [δ_H_ 6.40 (H-3'), 7.04 (H-6') and 6.37 (H-5')] [22]. Therefore, to the best of our knowledge, compound **19** was determined to be a new isoflavone and named cudracusisoflavone B.

#### Identification of known compounds

**Fig 4:**
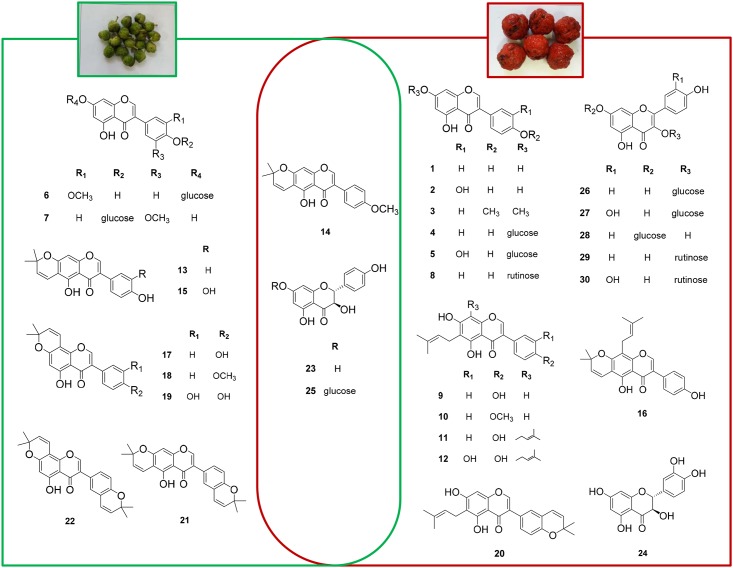
Chemical structures of compounds isolated from unripe and ripe fruits of *C*. *tricuspidata* fruits.

Twenty seven known compounds were identified, by the spectroscopic data analysis and comparison with literature values. These were genistein (**1**) [23], orobol (**2**) [24], 7,4'-dimethoxy-5-hydroxyisoflavone (**3**) [25], genistin (**4**) [26], oroboside (**5**) [27], 3'-*O*-methylorobol-7-glucoside (**6**) [28], sphaerobioside (**8**) [29], wighteone (**9**) [30], gancaonin A (**10**) [31], 4',5,7-trihydroxy isoflavonone (**11**) [32], 5,7,3',4'-tetrahydroxy-6-8-diprenylisoflavone (**12**) [33], alpinumisoflavone (**13**) [34], 4'-*O*-methylalpinumisoflavone (**14**) [32], 5,3',4'-trihydroxy-6'',6''-dimethylpyrano-[2'',3'';7,6]isoflavone (**15**) [33], scandenone (**16**) [34], derrone (**17**) [35], derrone-4'-*O*-methylether (**18**) [36], isochandalone (**20**) [37], ulexin B (**21**) [38], ulexone B (**22**) [39], (+)-dihydrokaempferol (**23**) [39], (+)-taxifolin (**24**) [40], (2*R*, 3*R*)-7-(β-glucopyranosyloxy)-2,3-dihydro-3,5-dihydroxy-2-(4-hydroxyphenyl)-4*H*-1-benzopyran-4-one (**25**) [41], astragalin (**26**) [42], hirsutrin (**27**) [43], populnin (**28**) [44], nicotiflorin (**29**) [45], and rutin (**30**) [45] (Fig 4).

#### Pancreatic lipase inhibitory effect

**Fig 5:**
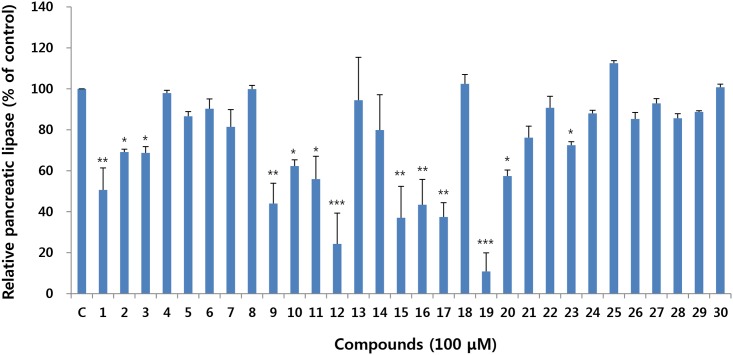
Effect of compounds on pancreatic lipase activity.

**Fig 6:**
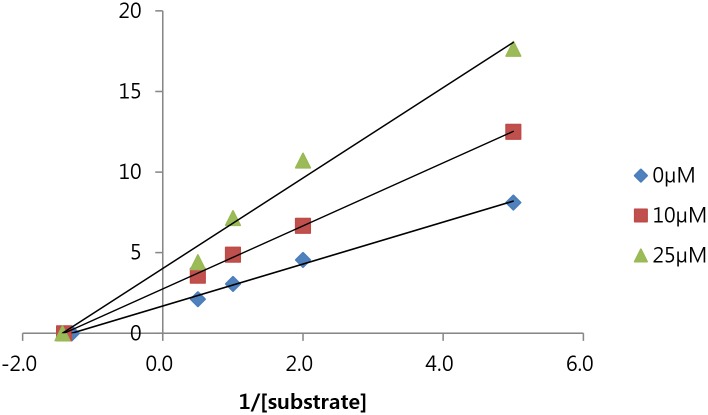
Lineweaver-Burk plots of the inhibitory activity of compound 19.

Pancreatic lipase inhibitory activity of isolated compounds was first determined employing in vitro assay system using porcine pancreatic lipase against *p*-NPB as a substrate. Among the compounds isolated from unripe and ripe fruits, compounds **1**, **9**, **12**, **15**, **16**, **17** and **19** showed >50% inhibition at 100 μM (Fig 5). In particular, a new compound from unripe fruits (**19**) showed the most potent inhibition with an IC_50_ value of 16.8 μM. Compound **19** also inhibited pancreatic lipase activity against triglyceride substrate with an IC_50_ value of 41.8 μM. For the further characterization of the mechanism of the inhibitory effect of compound **19** on pancreatic lipase, Lineweaver-Burk analysis was performed. As the concentration of compound **19** increased, the value for the *y*-intercept in the equation for each curve increased, whereas the *x*-intercept remained at a fixed point (Fig 6). These results suggest that compound **19** exerted an inhibitory effect on pancreatic lipase in a noncompetitive manner.

All the compounds isolated from *C*. *tricuspidata* fruits in this study belong to a class of flavonoids. They can be further subdivided according to the position of the phenyl moiety into flavonoids (2-phenylchroman) and isoflavonoids (3-phenylchroman). Compounds **1**–**22** are isoflavonoids and compounds **23**–**30** are flavonoids. Regarding structure-activity relationships, all the glycosides (**4**–**8** and **25**–**30**) showed weak activity, pointing to the importance of the absence of a glycoside moiety. The position and number of hydroxyl moiety also affected the inhibitory activity. The isoflavonoids that exhibited >50% inhibition (**1**, **9**, **12**, **15**–**17**, and **19**) have a free hydroxyl group at C-4'. The addition of a methoxyl group at C-4' (**3**, **10**, and **18**) resulted in lower inhibition (34.6, 39.8 and 22.5%, respectively) compared to that of corresponding isoflavonoids (**1**, **9** and **17**), which had stronger inhibitory activity (60.7, 65.7 and 68.2%, respectively). Isoflavonoids containing a 2,2-dimethylpyran ring at C-4' instead of a free hydroxyl group (**20**–**22**) showed weak inhibition (<30%). The number of hydroxyl groups also affected the inhibitory activity, as was observed by the stronger inhibitory activity (92.8 and 83.1%, respectively) the isoflavonoids with two hydroxyl groups at C-3', 4' (**12** and **19**), compared to that of its corresponding isoflavonoid with a hydroxyl group at C-4' (**11** and **17**) (31.5 and 68.2%, respectively). The findings showed that the position and number of the hydroxyl moiety, together with the presence of a glycoside moiety, affected the pancreatic lipase inhibitory activity of isoflavonoids from *C*. *tricuspidata* fruits.

#### Comparison of the compounds in the unripe and ripe fruits

The phytochemical investigation of *C*. *tricuspidata* revealed that xanthones were major constituents of the roots, whereas flavonoids were the major constituents of the fruits. Both xanthones of roots and flavonoids of fruits commonly contained isoprenyl moiety as side chains. They existed in diverse forms such as linear, cyclized or oxidized. The HPLC analysis of the unripe and ripe fruits extract revealed that isoflavonoids, with linear isoprenyl moieties, were major constituents of ripe fruits (**10** and **11**), whereas isoflavonoids with cyclized isoprenyl moieties (**13** and **17**) were the major constituents of unripe fruits. Further fractionation of the fruits resulted in the isolation of 12 compounds from unripe fruits and 21 compounds from ripe fruits. To better understand the difference in chemical composition between unripe and ripe fruits, isolated compounds were classified into two classes, compounds isolated only from unripe and compounds only from ripe fruits. Consistent with HPLC analysis for the major isoflavonoids, isoprenyl moieties were cyclized in unripe fruits, whereas exist in linear in ripe fruits. In addition, flavonoid glycosides are more abundant in ripe fruits. Although a more comparison is needed to clarify the chemical composition of *C*. *tricuspidata*, we cautiously suggest that the chemical composition of *C*. *tricuspidata* fruits depends on the maturation stages (Fig 4).

We recently reported the pancreatic lipase inhibitory activity of 6,8-diprenylgenistein, a major isoflavonoid of ripe fruits of *C*. *tricuspidata*. This compounds also showed anti-obesity effect in high-fat diet-induced obese mice [19]. Flavonoids are also known as potent antioxidants against oxidative stress which leads to the development of obesity and related complications. Therefore, flavonoids with pancreatic lipase inhibitory effect can be used as anti-obesity therapeutics by the combinatory action, which needs to be clarified with human study.

## Conclusions

We investigated the difference in the chemical composition of unripe and ripe fruits of *C*. *tricuspidata*. The unripe fruits had a higher content of total phenolics and flavonoids and exhibited stronger pancreatic lipase inhibition compared to the ripe fruits. HPLC analysis revealed the different chemical composition of the unripe and ripe fruits. Further fractionation resulted in the isolation of 30 compounds including two new isoflavonoids from the unripe fruits of *C*. *tricuspidata*. Analysis of the chemical composition of the unripe and ripe fruits revealed that 2,2-dimethylpyran ring, a cyclized prenyl was predominant in the unripe fruits, whereas a linear prenyl group in the ripe fruits. In addition, a new isoflavonoid from unripe fruits showed strong pancreatic lipase inhibition in a noncompetitive manner. Therefore, the maturation stage is an important factor in the maximum efficacy, and unripe fruits of *C*. *tricuspidata* are appeared to be a good source of new bioactive constituents for the regulation of obesity.

## Supporting information

S1 Fig ^1^H-NMR spectrum of compound 7 (MeOH-*d*_*4*_, 400 MHz). (DOCX)

S2 Fig ^13^C-NMR spectrum of compound 7 (MeOH-*d*_*4*_, 125 MHz). (DOCX)

S3 Fig HSQC spectrum of compound 7 (MeOH-*d*_*4*_, 125 MHz). (DOCX)

S4 Fig HMBC spectrum of compound 7 (MeOH-*d*_*4*_, 125 MHz). (DOCX)

S5 Fig ^1^H-NMR spectrum of compound 19 (Acetone-*d*_*6*_, 400 MHz). (DOCX)

S6 Fig ^1^H-NMR spectrum of compound 19 (MeOH-*d*_*4*_, 400 MHz). (DOCX)

S7 Fig ^13^C-NMR spectrum of compound 19 (Acetone-*d*_*6*_, 100 MHz). (DOCX)

S8 Fig HSQC spectrum of compound 19 (Acetone-*d*_*6*_, 100 MHz). (DOCX)

S9 Fig HMBC spectrum of compound 19 (Acetone-*d*_*6*_, 100 MHz). (DOCX)
