# Supplementary figures and images for: Comparison of pancreatic lipase inhibitory isoflavonoids from unripe and ripe fruits of Cudrania tricuspidata
Source: PLoS One. 2017 Mar 2;12(3):e0172069. doi: 10.1371/journal.pone.0172069 (PMC5333804; doi:10.1371/journal.pone.0172069)

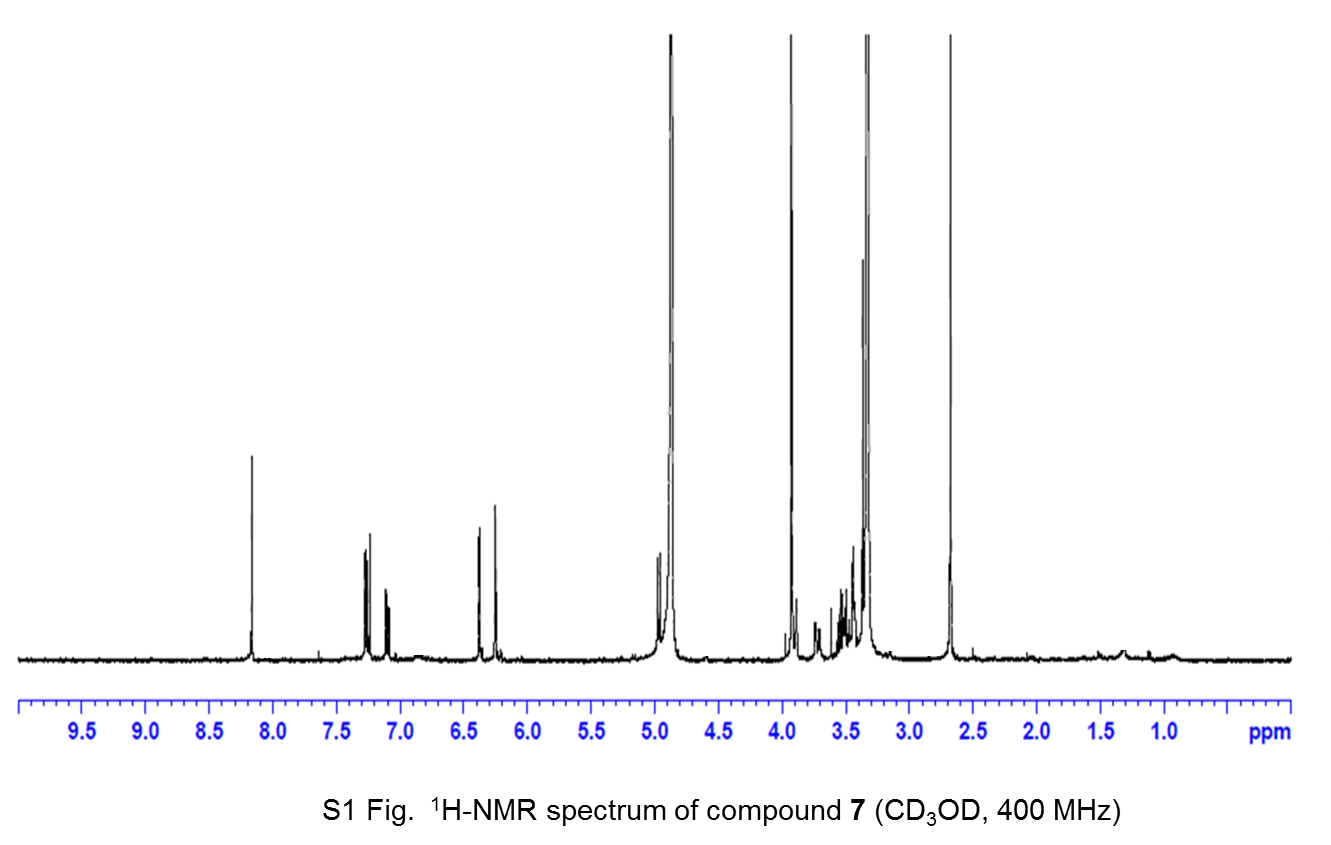

Supplement: S1 Fig — (DOCX) [file pone.0172069.s001.docx]

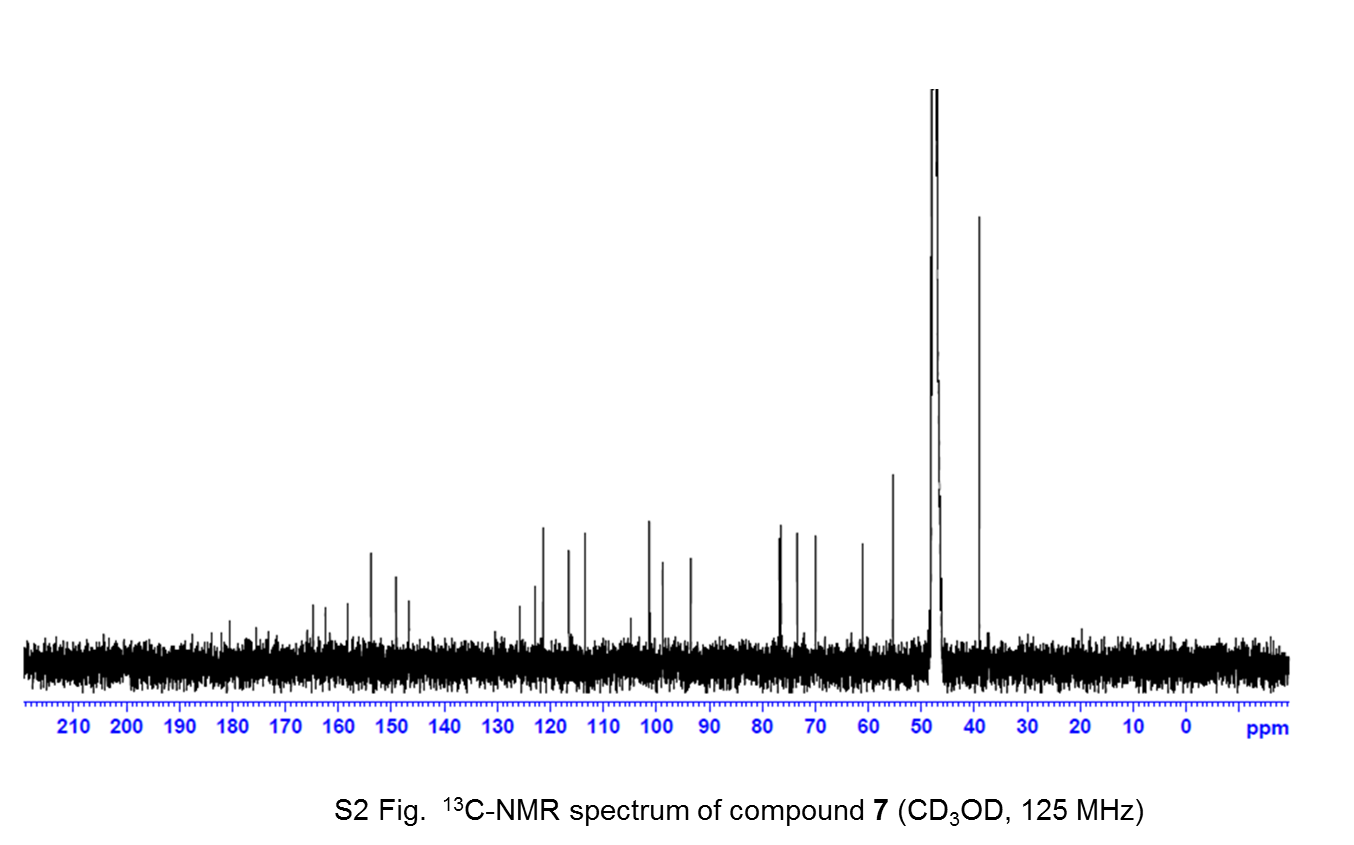

Supplement: S2 Fig — (DOCX) [file pone.0172069.s002.docx]

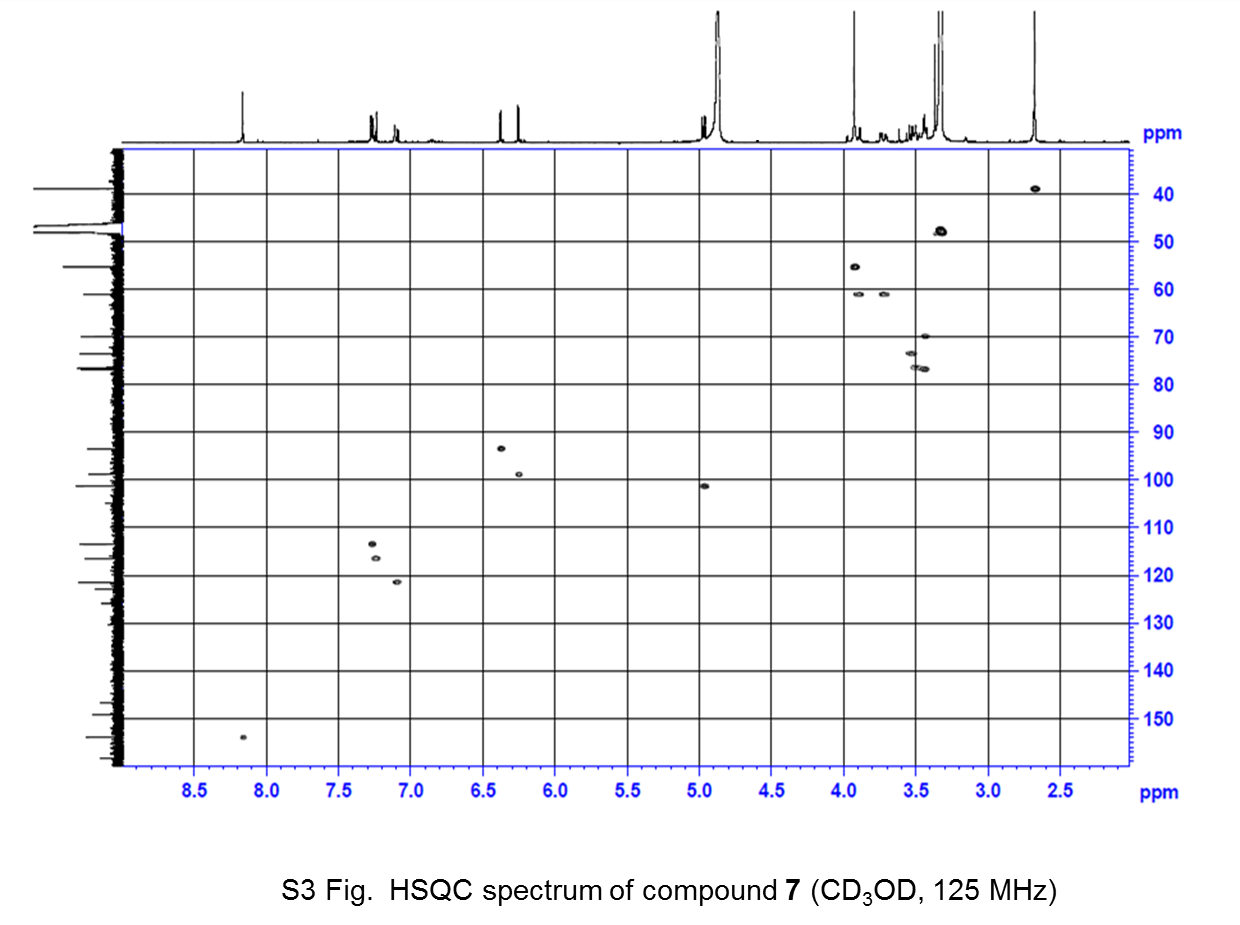

Supplement: S3 Fig — (DOCX) [file pone.0172069.s003.docx]

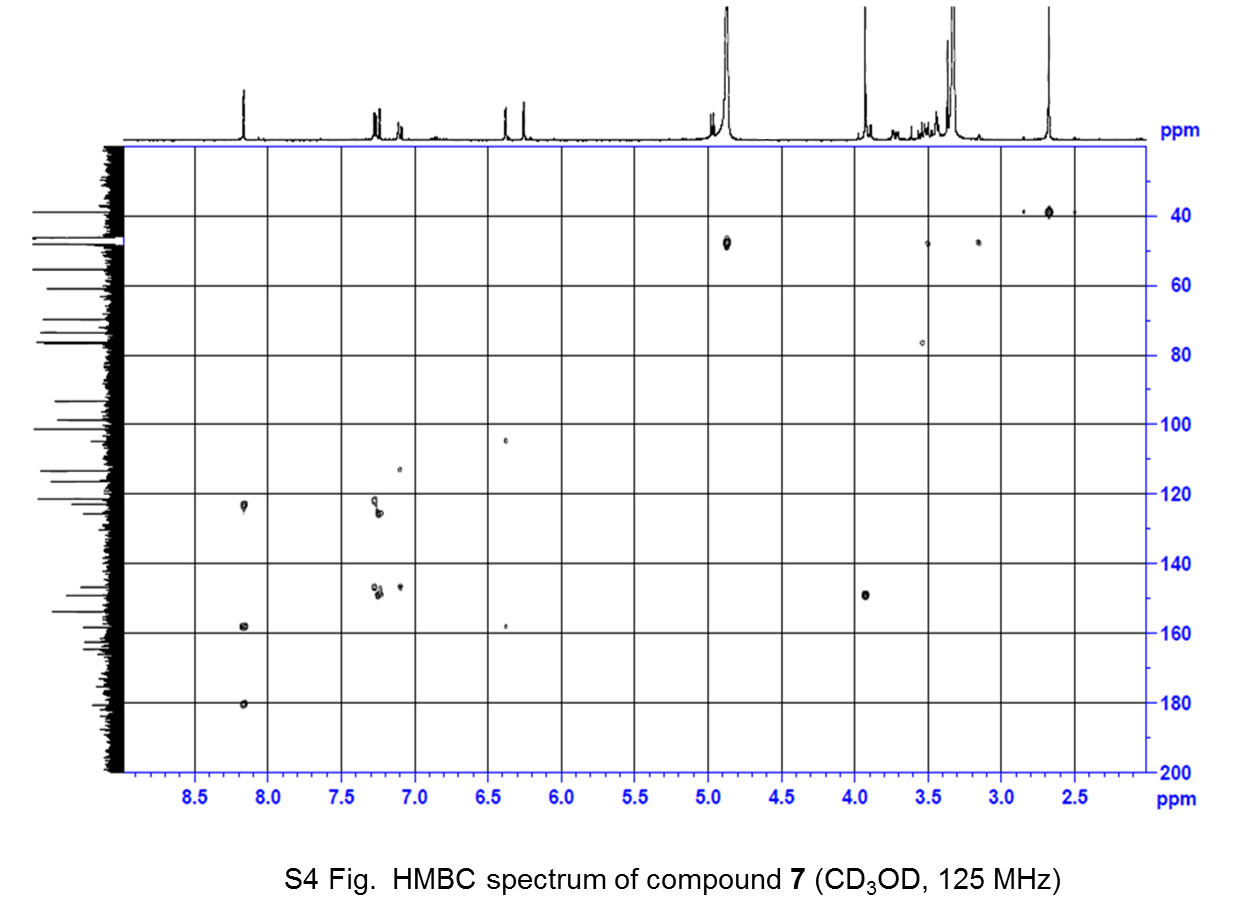

Supplement: S4 Fig — (DOCX) [file pone.0172069.s004.docx]

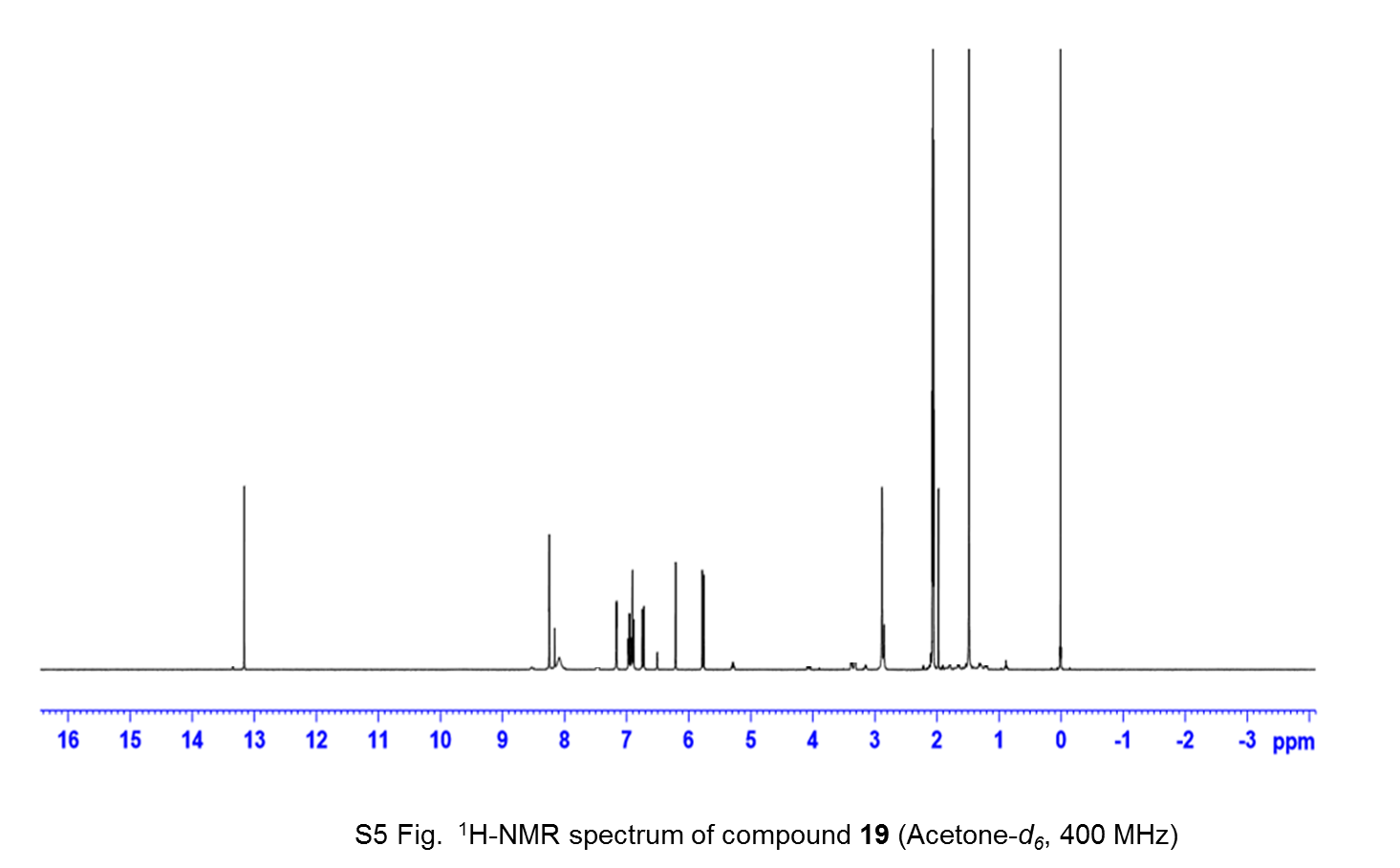

Supplement: S5 Fig — (DOCX) [file pone.0172069.s005.docx]

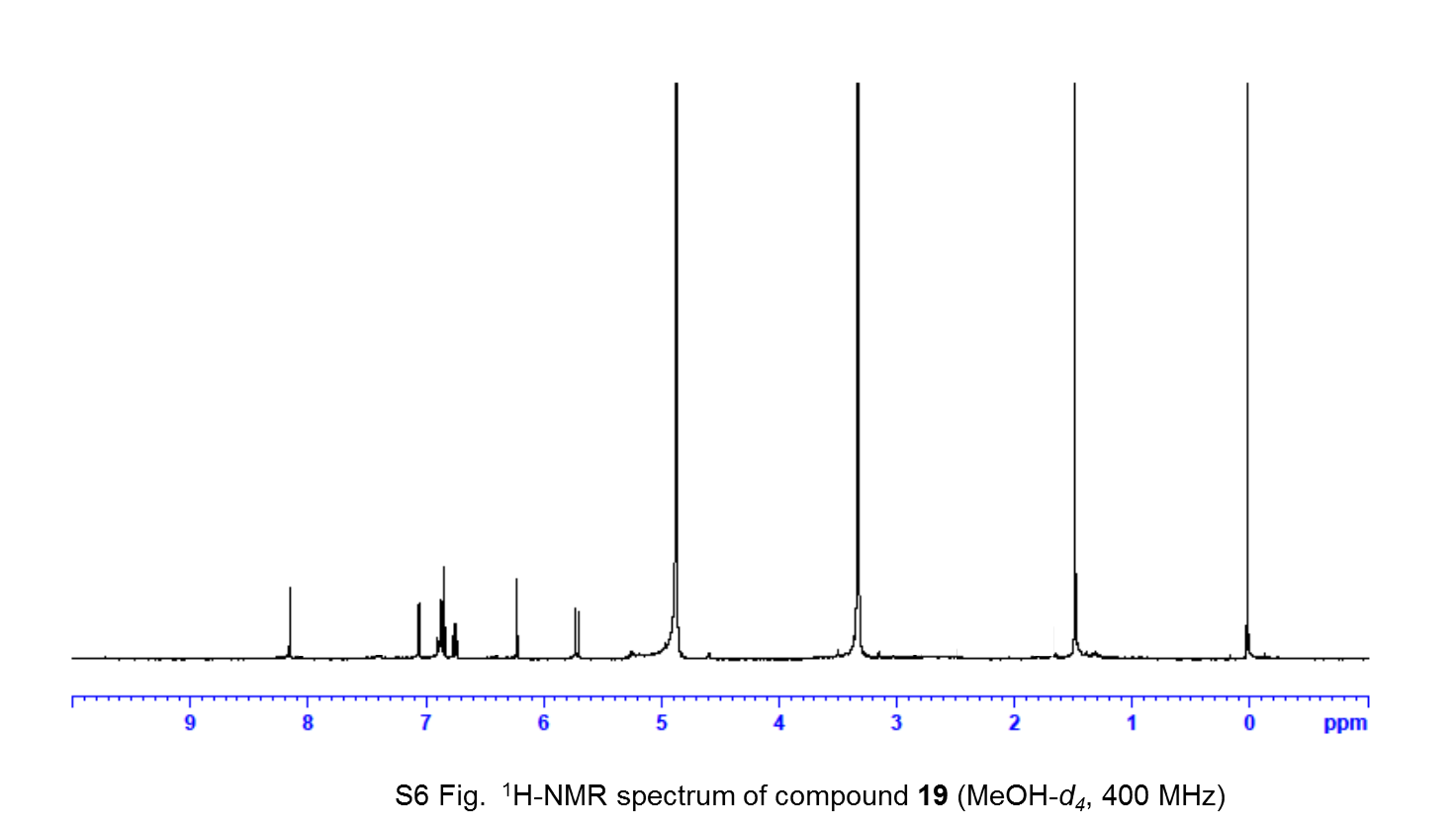

Supplement: S6 Fig — (DOCX) [file pone.0172069.s006.docx]

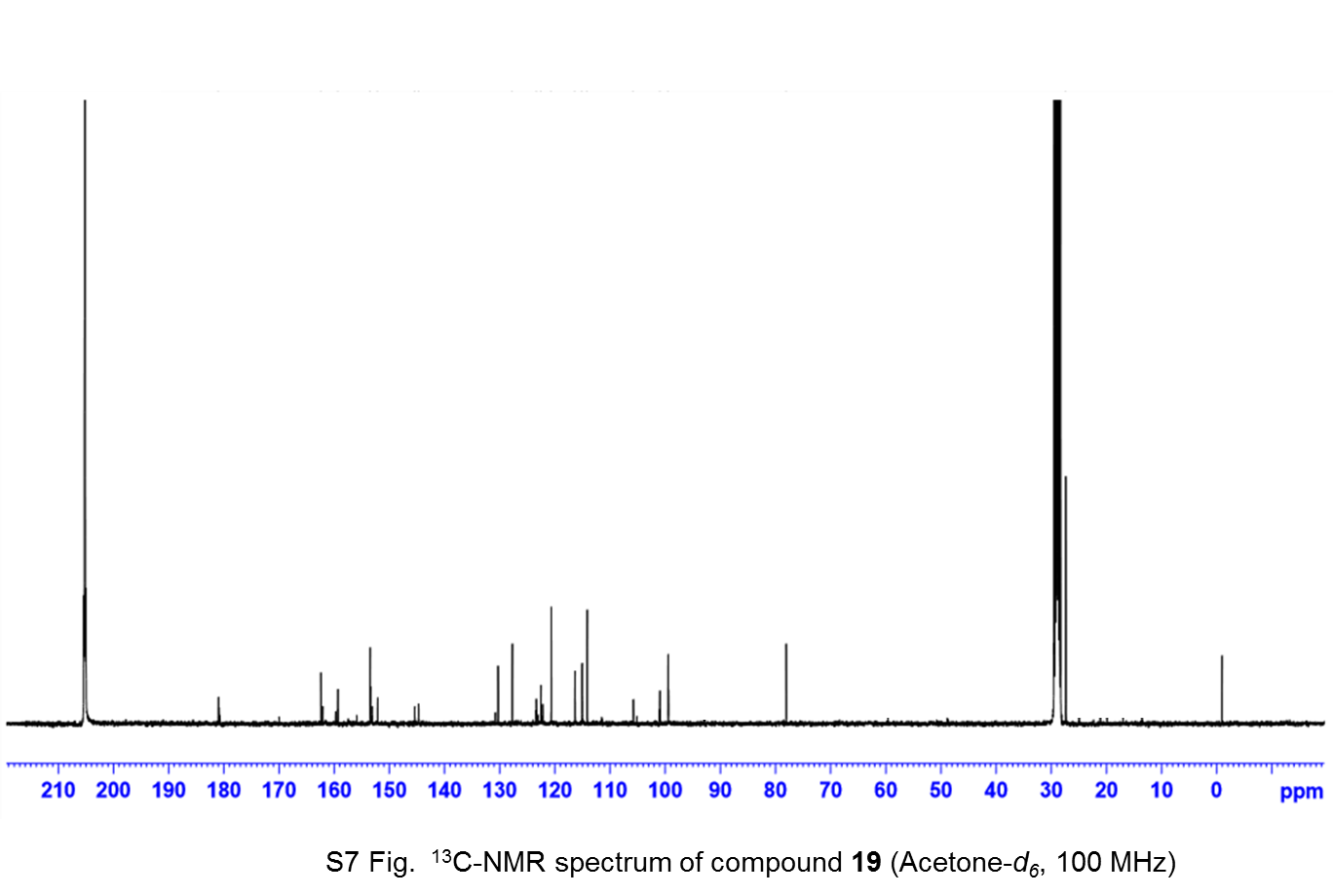

Supplement: S7 Fig — (DOCX) [file pone.0172069.s007.docx]

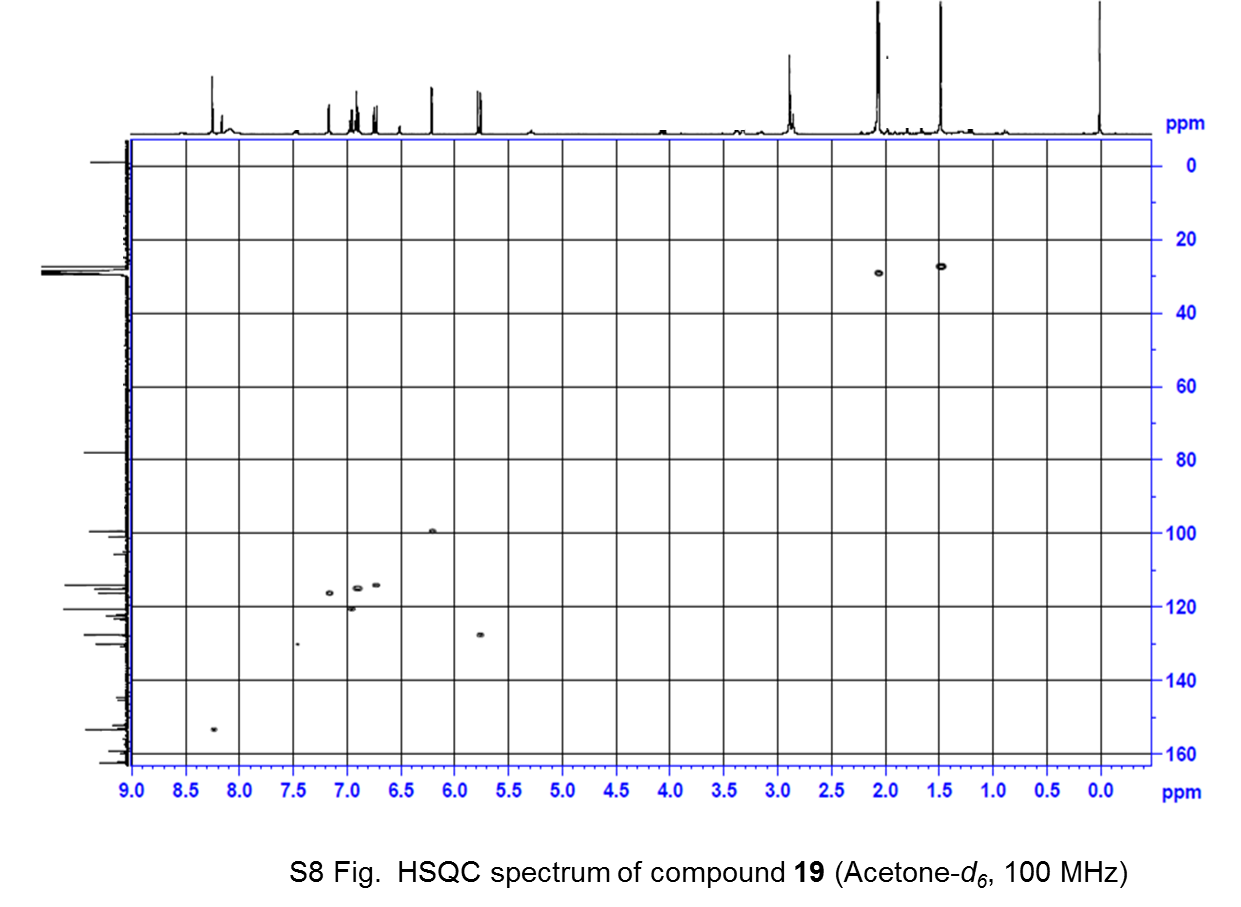

Supplement: S8 Fig — (DOCX) [file pone.0172069.s008.docx]

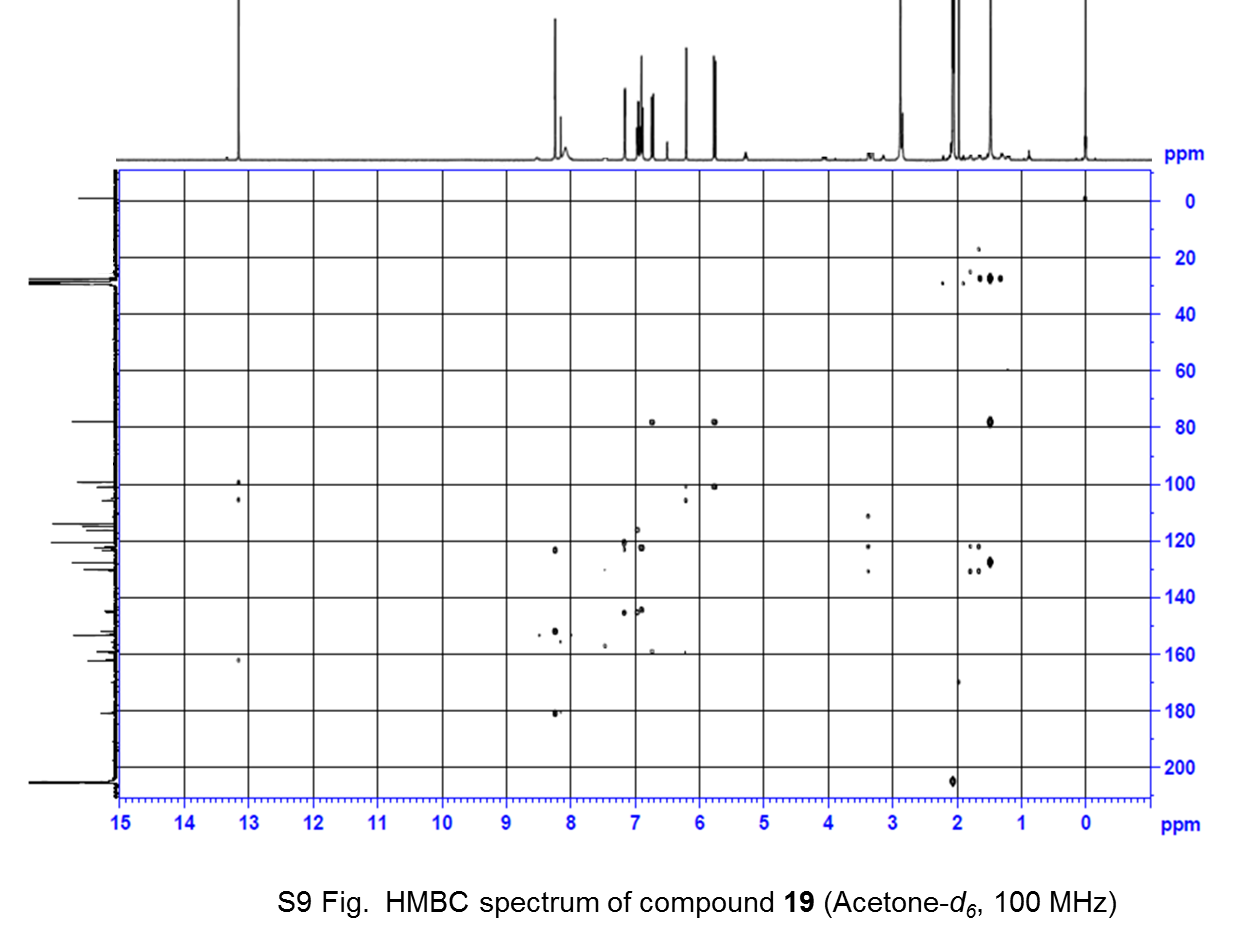

Supplement: S9 Fig — (DOCX) [file pone.0172069.s009.docx]
